# Comparison of serum 25-hydroxy vitamin D levels between mothers with small for gestational age and appropriate for gestational age newborns in Kerman

**Published:** 2015-04

**Authors:** Fatemeh Mirzaei, Tayebeh Amiri Moghadam, Peyman Arasteh

**Affiliations:** 1 * Department of Obstetrics and Gynecology, Physiology Research Center, Afzalipoor Hospital, Kerman University of Medical Sciences, Kerman, Iran..*; 2 * Department of Obstetrics and Gynecology, Afzalipoor Hospital, Kerman University of Medical Sciences, Kerman, Iran.*; 3 * Student Research Committee, Shiraz University of Medical Sciences, Shiraz, Iran.*

**Keywords:** *Small for Gestational Age*, *Appropriate for Gestational Age Pregnancy*, *Mother*, *Newborn*, *Vitamin D*

## Abstract

**Background:**

Vitamin D deficiency during pregnancy is associated with some adverse pregnancy outcomes but its relationship with fetal growth is unknown.

**Objective:**

We compared the 25-hydroxy vitamin D levels between mothers and their small for gestational age (SGA) newborns with mothers and their appropriate for gestational age (AGA) newborns.

**Materials and Methods:**

The study population included pregnant women that referred to Afzalipour Hospital in Kerman from 2012 to 2013. The case and control group consisted of 40 pregnant mothers with SGA and AGA newborns, respectively. The maternal and infants 25-hydroxy vitamin D levels were measured in the two groups.

**Results:**

25-hydroxy vitamin D deficiency (<20 ng/ml) was statistically higher in women with SGA newborns in comparison to women with AGA newborns (p=0.003).Vitamin D deficiency was higher among the SGA newborns in comparison to AGA newborns (25% vs. 17.5%), although this finding was not statistically meaningful (p=0.379). The relationship of vitamin D deficiency levels between mothers and infants in both the SGA group and the AGA group was significant.

**Conclusion:**

Our study reveals a high prevalence of vitamin D deficiency in women with SGA infants in comparison to women with AGA children. In addition, maternal vitamin D deficiency is associated with its deficiency in newborns.

## Introduction

Vitamin D deficiency is important in pregnancy because it has implications for both maternal and child health. Its deficiency is common in the general population, including pregnant women (1). Vitamin D plays an important role in many physiological functions like bone metabolism, cell function and reproduction. These functions are more prominent in women from their childhood to puberty and old age (2). An adequate 25-hydroxy vitamin D (25-OH-D) level is determined as ≥32 ng/ml. Vitamin D insufficiency and deficiency are diagnosed as levels less than 32 ng/ml and 20 ng/ml respetively (3). Low levels of vitamin D are related to some adverse outcome of pregnancy such as premature delivery, bacterial vaginosis, hypertension, and gestational diabetes. Caesarian section is four times more common among women with low levels of 25-OH-D than women with normal levels of vitamin D (4-9). Vitamin D deficiency during pregnancy may also have a relation with the risk of developing small for gestational age (SGA) newborns (10-13). In general, 4-7% of all born infants in developed countries are SGA. This prevalence depends on the population under study, geographical concentration and the standard curve used as reference for assessing infants (14, 15). 

Fetal growth retardation is the main cause for death and neonatal complications. The prenatal death rate increases by 1.5 or 2 folds in infants and fetuses with delayed growth (16). Some studies recently reported an association between low maternal 25-OH-D levels and preeclampsia and gestational diabetes but there is little evidence to evaluate the effect of vitamin D status on SGA (1, 17, 18). The aim of the study was to compare the 25-hydroxy vitamin D levels between mothers and their SGA newborns with mothers and their appropriate for gestational age (AGA) newborns.

## Materials and methods

**Sampling and data collection**

In this case control study, all mothers who referred to Afzalipour Hospital, Kerman, Iran from spring 2012 to winter 2013 randomly evaluated. The study protocol was approved by the Ethics Committee of Kerman University of Medical Sciences. All the mothers in the study delivered in their third trimester. The case group consisted of 40 pregnant women with SGA infants and the control group consisted of 40 pregnant women with AGA newborns. All the participants filled a consent form to enter the study.

Information regarding age, parity, weight before pregnancy, height, multivitamin consumption during pregnancy, and gestational age at was registered. The exclusion criteria included: infants with congenital anomalies at birth, multiple pregnancies, intrauterine fetal death, maternal chronic hypertension, preeclampsia, diabetes, and drug addiction. The mothers’ serum and the infants’ umbilical cord samples were taken, centrifuged and preserved in -80^o^C. Serum concentration of 25-OH-D was measured using the Enzyme-Linked Immunosorbent Assay (ELISA) method.

**Definition of variables**

SGA was defined as the fetal weight less than the 10^th^ percentile for gestational age and was diagnosed via ultrasonography. AGA diagnosis was considered as the fetal weight between the 10-90^th^ percentile for gestational age. In both groups in case that the birth weight was not consistent with the sonography weight estimations, the women were removed from the study. Vitamin D deficiency was regarded as levels less than 20 ng/ml and levels between 20-32 ng/ml were considered as insufficient, and levels ≥32 ng/ml were regarded as adequate (3).

**Statistical analysis**

Statistical data analyses were done using the SPSS Data were analyzed using Student’s *t* test, chi-square test, and logistic regression. A two-tailed p˂0.05 was considered as statistically meaningful.

## Results

The mean age in the control group was 26.87±4.60 years and in the case group was 26.78±5.26 years. Overall the two groups consisted of 43 nulliparous women. Four people (5%) had a BMI less than 18.5 and 3 people (3.75%) had a BMI more than 30. The two groups did not show a statistically meaningful difference in age, parity, and body mass index (BMI). 

Multivitamin consumption was statistically higher among mothers who had AGA newborns (75% and 45% in the AGA and SGA group, respectively; p=0.021). The pregnancy age at delivery time did not display a significant difference between the two groups (37.10±1.05 weeks and 37.32±0.94 weeks in the AGA and SGA groups, respectively; p=0.864). The birth weight in the control group was 2998.5±55g and in the case group it was 2115±33.76g (p=0.001) (Table I).

The prevalence of vitamin D deficiency in women with SGA newborns was 45%, while only 20% of women in AGA group had vitamin D deficiency (p=0.003). Although the vitamin D level was higher among the AGA newborns, the results did not display a statistically significant difference among the two groups in overall vitamin D levels (70% and 55% in the AGA and SGA newborns had sufficient vitamin D levels, respectively; p=0.379) (Table II).

The maternal BMI and age in both groups had no relation with vitamin D deficiency in the mothers (p=0.443 and p=0.721 for maternal age, p=0.231 and p=0.847 for BMI in the SGA and AGA group, respectively). The relation between the use of multivitamins and vitamin D deficiency in mothers in both groups was not significant (p=0.585 and p=0.156 in the SGA and SGA groups, respectively). The relationship between vitamin D deficiency in mothers of both groups and its deficiency in the cord blood samples was significant (p=0.026 and p=0.009 in the SGA and AGA groups, respectively) (Table III).

**Table I:** Comparing mothers and infants characteristics in both study groups (n=40)

**Variables**				**Case group**	**Control group**	**p-value**
Maternal age (Years)*				26.78 ± 5.26	26.87 ± 4.60	0.906#
Number of births**						
		1		24 (80)	19 (48.7)	0.148##
		≥2		16 (20)	21 (51.3)	
BMI (Kg/m^2^)**						
	<18.5			3 (7.5)	1 (2.5)	0.380##
	18.5-24.9			28 (70)	33 (82.5)	
	25-29.9			8 (20)	4 (10)	
	≥30			1 (2.5)	2 (5)	
Gestational age at delivery (Weeks)*				37.32 ± 0.94	37.10 ± 1.05	0.864#
Birth weight (g)*				2115 ± 3.76	2998 ± 5.55	0.001#
Multivitamin user**						
			Yes	18 (45)	30 (75)	0.021##
			No	22 (55)	10 (25)	

* Data are presented as mean±SD.

** Data are presented as n (%)

# Student’s *t *test,

## chi-square test

**Table II:** Comparing vitamin D levels in mothers and the newborns of both groups

**25-hydroxy vitamin D levels (ng/ml)** *		** Case group** **n(%)**	** Control group** **n(%)**	**p-value**
**Maternal**				
	<20	18 (45)	8 (20)	0.003
	20-32	12 (30)	7 (17.5)	
	≥32	10 (25)	25 (62.5)	
**Newborns**				
	<20 ng/ml	10 (25)	7 (17.5)	0.379
	20-32 ng/ml	8 (20)	5 (12.5)	
	≥32 ng/ml	22 (55)	28 (70)	

BMI: Body mass index

Chi-square test

* Deficiency: <20 ng/ml, Insufficiency: 20-32 ng/ml, Sufficiency: ≥32 ng/ml.

**Table III:** The relationship between vitamin D deficiency in mothers with maternal age, BMI, multi vitamins use, and vitamin D deficiency in newborns

**Maternal vitamin D deficiency**	**Case group- OR (CI 95%)**	**p-value**	**Control group- OR (CI 95%)**	**p-value**
Maternal age (Years)	0.932 (0.778-1.11)	0.443	1.03 (0.875-1.21)	0.721
BMI ((Kg/m^2^)	0.312 (0.046-2.14)	0.237	0.853 (0.169-4.31)	0.847
Number of multivitamin users	0.569 (0.075-4.29)	0.585	5.50 (0.51-58.31)	0.156
Newborn 25(OH) D levels <20 (ng/ml)	2.43 (1.57-14.55)	0.026	2.36 (2.20-25.24)	0.009

BMI: Body Mass Index

logistic regression

## Discussion

Our study reveals a high prevalence of vitamin D deficiency in women with SGA infants in comparison to women with AGA children. Vitamin D deficiency is a common 

issue worldwide. In most countries, there is no routine monitoring of serum 25-OH-D levels during pregnancy. The prevalence of vitamin D deficiency during pregnancy is different according to the population of the study (18). The documented prevalence is 31% in India, 19.5% in Greece and 5.7% in Iran (19-21). SGA is among the main causes of death and prenatal complications and its etiology is multi-factorial (14-16). The placenta and maternal decidual cells are the places that active vitamin D, 1-25 dehydroxy vitamin D3 are synthetized (22). It has been shown that 1-25 dehydroxy vitamin D3 increases the synthesis of the vascular endothelial growth factor. A probable mechanism for the role of vitamin D in fetal growth is through its probable function in the placenta (23, 24).

There are few studies that have evaluated the relationship between vitamin D and fetus weight and growth. In a study by Gernand *et al* vitamin D levels less than 37 nmol/I in the first three months were associated with an increased risk of SGA occurrence (OR=0.5, 95% CI: 0.3-0.9, p=0.01) but this relationship was not observed in the second trimester (1). This finding is similar to the study by Bodnar *et al* in which they documented a relationship among the vitamin D levels in mothers in the first trimester with the risk of SGA in Caucasian women, but this relation was not observed among black women (9).

In our study 45% of the women in the SGA group and 20% of women in the AGA group had vitamin D deficiencies. Moghbeli *et al* reported that the prevalence of vitamin D deficiency is 66.8% (35< ng/ml) in pregnant women in Iran (25). In their study pregnant women were included regardless of their birth weight; furthermore their geographical region of study and the cutoff point of vitamin D deficiency were different in comparison to ours. There was no significant association between vitamin D deficiency and BMI and maternal age. Similar result was reported by Yasser *et al* (26).

In our study 25% of the cord blood samples and 17.5% in the AGA group had vitamin D levels that were less than 20 ng/ml. In a study by Hitrova *et al* the vitamin D levels were evaluated in mothers and newborn with low birth weight (27). They reported that 62.5% of mothers and 38.6% of newborns had vitamin D deficiency (<20 ng/ml). In comparison to our study, the frequency of vitamin D deficiency was more in both the mothers and cord blood samples. This study was similar to our study regarding its cutoff point for consideration as vitamin D deficiency (<20 ng/ml).

We found a significant relation in vitamin D deficiency between mothers and infants in both the SGA and AGA groups which is similar to the findings of Hitrova *et al* and Moghbeli *et al* (25, 26). 75% of women in our study in the AGA group and 45% of women in the SGA group were taking multivitamins during their pregnancy. In our study the multi vitamin consumption in the AGA group was more but no significant relation was documented between vitamin D deficiency in mothers and the multivitamin consumption. 

Marya RK, et al study have demonstrated third trimester vitamin D supplement to reduce of SGA and low birth weight (28). In our investigation relationship between the use of multivitamin and vitamin D deficiency in mothers in both groups was not significant. Some studies found improved vitamin D levels using multivitamin supplements. In two studies using multivitamin supplements were more effective than sunlight in treating vitamin D deficiency (29, 30). In the study by Song *et al* pregnant women using multivitamin supplements had higher vitamin D levels than the patients who did not use it (32.3±9.5 ng/ml, vs. 24.9±8.2 ng/ml; p<0.001) (18). 

## Conclusion

Our study shows a high prevalence of vitamin D deficiency in women with SGA infants in comparison to women with AGA children and also we found a significant relation in vitamin D deficiency between mothers and infants in both the SGA and AGA groups. Additional Studies are needed to relationship of vitamin D deficiency and SGA. 
